# Cardiac Output Assessed by Invasive and Minimally Invasive Techniques

**DOI:** 10.1155/2011/475151

**Published:** 2011-07-06

**Authors:** Allison J. Lee, Jennifer Hochman Cohn, J. Sudharma Ranasinghe

**Affiliations:** Jackson Memorial Hospital, University of Miami, Miami, FL 33136, USA

## Abstract

Cardiac output (CO) measurement has long been considered essential to the assessment and guidance of therapeutic decisions in critically ill patients and for patients undergoing certain high-risk surgeries. Despite controversies, complications and inherent errors in measurement, pulmonary artery catheter (PAC) continuous and intermittent bolus techniques of CO measurement continue to be the gold standard. Newer techniques provide less invasive alternatives; however, currently available monitors are unable to provide central circulation pressures or true mixed venous saturations. Esophageal Doppler and pulse contour monitors can predict fluid responsiveness and have been shown to decrease postoperative morbidity. Many minimally invasive techniques continue to suffer from decreased accuracy and reliability under periods of hemodynamic instability, and so few have reached the level of interchangeability with the PAC.

## 1. Cardiac Output Assessed by Invasive and Minimally Invasive Techniques

Cardiac output (CO) measurement has long been considered essential to the assessment and guidance of therapeutic decisions in critically ill patients, by providing an indirect indication of systemic oxygen delivery and global tissue perfusion. Perioperatively, CO monitoring has become virtually routine for certain high-risk patients and in major surgeries, where large fluid shifts are expected.

## 2. History

The technique was first described in 1870 by Adolf Fick [1], who computed an animal's CO by utilizing oxygen concentrations in arterial and venous blood samples, where CO is equal to oxygen consumption (VO_2_), divided by arterial oxygen content (CaO_2_) minus mixed venous oxygen content (CvO_2_) [2, 3]:

(1) $$\text{CO}=\frac{{\text{VO}}_{2}}{\left(\text{Ca}{\text{O}}_{2}-\text{Cv}{\text{O}}_{2}\right)\times 10}.$$

Pulmonary artery catheterization was first performed experimentally in dogs by Grehant and Quinquaud in 1886, but the technique would not become available to humans for another fifty years [4]. 

Indicator-dilution techniques later developed. In 1897, Stewart described experiments pioneering the indicator-dilution principle, when he injected sodium chloride into the central circulation of animals and measured its subsequent concentration in the femoral artery [5]. Hamilton modified this principle to account for the varying concentrations of diluted injectate over time in human circulation, developing a time concentration curve to reflect this phenomenon [2]. CO was shown to equal the quantity of indicator dye (indocyanine green) injected, divided by the area under the dilution curve measured downstream, today known as the Stewart-Hamilton equation [5]:

(2) $$\text{Flow}=\frac{C_{0}V_{0}}{\int c\left(t\right)\text{d}t},$$

where *C*_0_ denotes initial injectate concentration and *V*_0_ represents initial injectate volume. The denominator represents the concentration of diluted injectate over time, thus the area under the dilution curve.

Based on the same concept as indicator-dilution methods, Fegler introduced thermodilution (TD) in 1954 by injecting a cold solution as an indicator and measuring changes in blood temperature detected distally [6]. In 1970, Swan et al. developed what they termed a “flow-directed balloon-tipped” multiple lumen catheter, the pulmonary artery catheter (PAC) [7]. The introduction of the PAC enabled physicians to measure CO by TD both at the bedside and intraoperatively. Forty years later, this method is still considered the clinical gold standard for CO measurement, secondary to its extensive utilization in a variety of clinical settings.

## 3. Intermittent Bolus Pulmonary Artery Thermodilution

**Figure 1:**
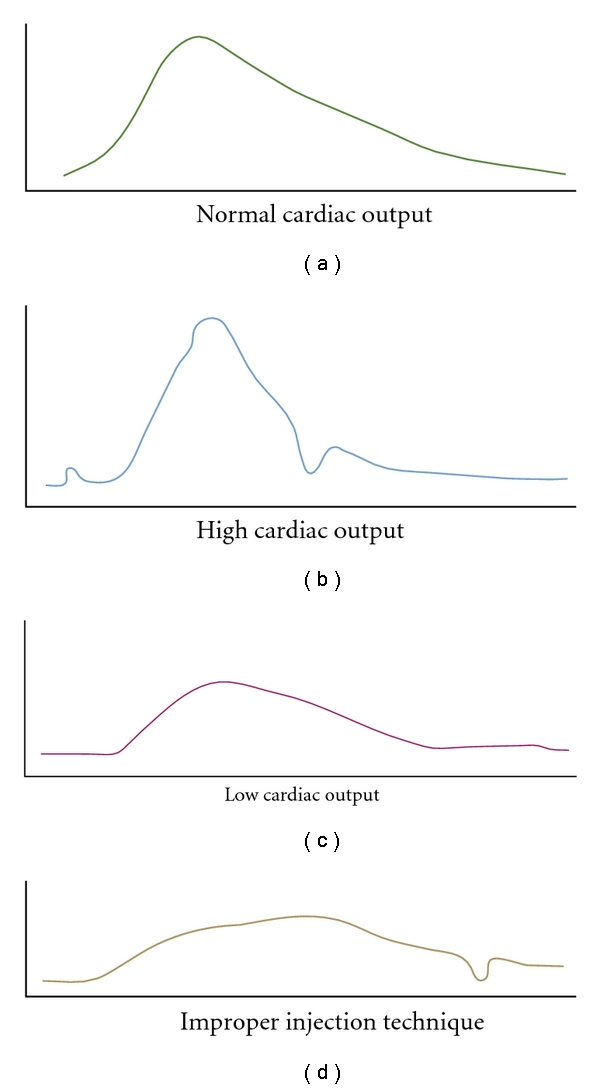
Thermodilution cardiac output curves. Used with permission from [8].

The TD technique is founded on the law of conservation of energy [9]. A known amount of cold solution is injected through the proximal port of a PAC into the right atrium, and this solution is detected distally by a thermistor several centimeters from the end of PAC. The change in blood temperature detected causes a change in the thermistor resistance, allowing for the calculation of the area under the TD curve. CO is determined from a modified Stewart-Hamilton equation [10, 11]:

(3) $$\text{CO}=\frac{\mathrm{VI}\;*\left(\text{TB}-\text{TI}\right)*K1*K2}{\int \Delta \text{TB}\;\left(t\right)\text{d}t},$$

where *VI*  is injectate volume, TB is blood temperature, TI is injectate temperature, *K*1 is a density factor: (specific heat (injectate) × specific gravity (injectate))/(specific heat (blood) × specific gravity (blood)), and *K*2 is a computation constant accounting for heat exchange in transit, injection rate, and catheter dead space. The denominator, change of blood temperature as a function of time, reflects the area under the TD curve (Figure 1) [6].

## 4. Reliability of Thermodilution

Despite being considered the gold standard technique for CO measurement, the reproducibility of TD technique has been heavily scrutinized and, to our knowledge, no data on the subject has been published in the last 20 years. Studies continue to quote statistical significance as demonstrated by Stetz et al. in 1982 [12], where the accuracy of TD was compared to that of Fick and dye-dilution methods. The conclusion was that all three methods are “of equal merit.” The intrinsic reproducibility of TD measurements was also analyzed, with the conclusion being that there must be a minimal difference of 12–15% between three serial CO determinations, to suggest clinical significance [12]. The inherent error of TD measurement, has subsequently been quoted in this manner.

### 4.1. Sources of Error

Accurate CO estimation can only be made if several assumptions are true. The amount of cold injectate must remain constant between the injection and detection sites. There must be complete mixing of blood and injectate and no fluctuations in baseline blood temperature during measurement [9]. Sources of error may be considered to be technical or intrinsic to certain physiologic states.

Technical errors can be due to loss of indicator before, during, or after injection, variability of temperature and volume of injectate, and thermistor malfunction. Although TD was first performed with 10 mL of iced 5% dextrose water, most studies over time have demonstrated no difference in accuracy when iced or room-temperature injectate was used [2, 10, 13]. When using an iced indicator, rewarming of injectate prior to administration and heat transfer during transit can both result in an overestimation of CO. When the volume of indicator injected is less than the assumed amount, an overestimation of CO can occur. Recommended volumes are 10 mL for adults and 0.15 mL/kg for children [6]. A clot over the catheter tip or contact with a vessel wall due to a wedged catheter can insulate the thermistor and result in spurious measurements. An injection time of 4 seconds or less with steady pressure has been recommended to prevent a delayed upstroke of the TD curve. Coiling of the catheter may change the distance from the injection site to the tip and also introduce error [6]. 

Both physiologic and pathologic states can lead to inaccurate CO measurements. Fluctuations in baseline pulmonary artery temperature occur with cardiac and respiratory oscillations. Rewarming in the initial minutes after cardiac bypass results in a transient decrease in core body temperature as heat distributes to the periphery. Measurements taken at this time can significantly underestimate the true CO [14]. Simultaneous rapid intravenous infusions have also been shown to alter computed CO [2].

It should be emphasized that TD with a PAC measures right ventricular outflow and not systemic CO. Intracardiac shunts can, therefore, lead to inaccurate measurements. In patients with left-to-right shunts, early recirculation of injection results in a subsequent distortion of the downward slope of the TD curve [15]. In the presence of right-to-left shunts, a portion of the indicator will bypass the thermistor, resulting in an overestimation of CO. Both pulmonary and tricuspid valve insufficiencies can likewise lead to unreliable CO determinations. The recirculation of indicator across incompetent valves can overestimate or underestimate CO, depending on the severity of the regurgitation and the underlying systemic CO [2]. 

Spontaneous and mechanical ventilation both alter right ventricular output throughout the respiratory cycle more so than left ventricular outflow. Studies evaluating the effects of the mechanical ventilatory cycle on TD measurements reveal inspiratory decreases in right ventricular ejection fraction and subsequent increases in right ventricular end systolic volumes [16]. A fall in left-sided CO, however, is largely prevented by the increase in right ventricular end diastolic volume. These findings explain the greater ventilatory modulation of right ventricular volumes. In the past, measurements taken at the end of expiration were thought to produce the greatest reproducibility. On the contrary, it is argued that more reliable estimations of mean TD CO should be taken from three to four serial CO measurements at different phases of the cycle [6]. Some authors recommend at least eight measurements taken randomly at different times during the ventilatory cycle [16].

## 5. Continuous Pulmonary Artery Thermodilution

Applying the same principles of TD, newer technologies applied to PACs allow continuous CO measurements. By utilizing an electric filament incorporated into the right ventricular portion of the PAC, blood flowing through the right heart is heated intermittently, approximately 15 to 25 cm proximal to the PAC tip. The resulting thermal signal is measured by a thermistor at the catheter tip. CO measurements by continuous TD have been shown to generally correlate well with intermittent bolus measurement [2]. These catheters provide a continuous trend of CO, decrease operator workload, and possibly reduce infection risk associated with bolus technique. However, since the values displayed are updated every 30 to 60 seconds, what is reflected is the average value for CO measured over the previous 5–15 minutes [9]. Leibowitz and Oropello [17] studied average in vivo time delays associated with sudden changes in CO of critically ill patients. The mean in vivo response times were reported to be 9.3, 10.5, and 11.8 minutes for a 50, 75, and 90% response, respectively [17]. Due to these inherent time delays, many clinicians argue these continuous monitors should be considered a “continual” rather than continuous real-time monitor [15]. These catheters may therefore be less optimal in detecting and measuring abrupt CO changes, but could be a more accurate representation of global CO.

### 5.1. Controversies Regarding Use

In the early 1980's, studies demonstrated improved outcomes with both perioperative and intensive care utilization of pulmonary artery catheterization [17]. However, in 1987, Gore et al. [18] published a study showing that mortality from myocardial infarction actually increased with PAC use. Although this investigation was merely a case-control, chart review study without retrospective risk adjustment, the article led to an editorial calling for a “moratorium” on PAC use [19]. 

Studies using the PAC to optimize cardiac index, mixed venous oxygen saturation (Sv02), and oxygen delivery have failed to show any reduction in morbidity and mortality of critically ill patients. In the large multicenter, SUPPORT study, a propensity score using multivariable logistic regression, looked at the association between right heart catheterization and specific outcomes. Investigators revealed an increase in 30-day mortality in patients with PACs [20]. The National Heart, Lung, and Blood Institute (NHLBI) and Food and Drug Administration (FDA) have published a consensus statement advocating for RCTs with the PAC in patients with congestive heart failure (CHF), acute respiratory distress syndrome (ARDS), sepsis, and septic shock, as well as low-risk coronary artery bypass graft surgery (CABG) [21].

### 5.2. Evidence from Randomized Controlled Trials

In 2003, the Canadian Critical Care Clinical Trials Group reported the largest RCT to date, comparing goal-directed therapy using a PAC versus standard care with a central venous catheter (CVC) [22]. There were no differences in hospital mortality, median length of stay (LOS), or one-year survival rates, despite an increased use of inotropic agents, vasodilators, antihypertensives, and erythrocyte and colloid transfusions in the PAC group. PAC-related adverse events occurred in 1.5% of patients versus 0.7% related to central venous catheter use alone [22].

In 2005, the PAC-Man study, a RCT done in United Kingdom ICUs, also failed to show evidence of benefit or harm with PAC management [23]. The LOS in the ICU and hospital and days of organ support required were similar in patients managed with and without a PAC. There was a 10% (46 in 486) incidence of direct complications due to PAC use, the most frequent being hematoma formation, arterial puncture, and arrhythmias. This study, similar to Sandham et al. [22], refuted the claim of increased mortality associated with catheter use. Other studies utilizing a PAC in patients with severe sepsis, septic shock, and/or ARDS failed to show a change in mortality rate [24–26]. In 2006, low-risk patients undergoing off-pump, beating-heart surgery showed no difference in operative mortality or outcome variables between patients with or without PACs [27].

The ESCAPE trial, funded by the NHLBI, evaluated the effectiveness of PACs guiding therapy in patients with severe CHF [28]. The use of the PAC had no effect on the primary endpoint of days alive out of hospital; however, a trend for “greater functional improvement after therapy guided by the PAC” was reported [28]. A concurrent PAC registry was established for hospitalized heart failure patients with a PAC who were not randomized to the trial. The study has been criticized for excluding patients with a higher disease severity and mortality risk [29]. In addition, no treatment protocol or proven therapy was directed towards PAC use [30]. Due to a lack of goal-directed therapy, both groups of patients likely received similar interventions.

A RCT conducted by the NHLBI Acute Respiratory Distress Syndrome (ARDS) Clinical Trials Network in 2006 compared treatment of acute lung injury in patients managed with PACs versus CVCs [25]. There were no statistical differences in mortality in the first 60 days before discharge home, ventilator free days, or LOS in ICU. The PAC group received more red blood cell transfusions and had approximately twice as many catheter-related complications, most commonly arrhythmias.

Why is it that, despite such detailed hemodynamic information, PACs fail to improve patient outcomes? One suggestion is that the lack of goal-directed therapy tailored towards PAC use has prevented us from altering morbidity and mortality. Pulmonary artery catheterization should be seen as a diagnostic tool and not mistaken to be therapeutic [31].

## 6. Minimally and Noninvasive Techniques

Although TD may be considered the gold standard for CO measurement, its use is limited, mainly because of the associated risks of pulmonary artery catheterization (arrhythmias, valvular lesions, infection, pulmonary emboli, pulmonary infarction, and pulmonary artery rupture). Additional costs are also significant. The ideal technique for CO measurement is minimally or noninvasive, is continuous, does not require calibration, and is accurate, reproducible, and reliable during various physiologic states [32]. A multitude of new technologies for CO measurement have been developed and are now available for clinical use (Table 1).

### 6.1. Methods of Comparison

Studies of reliability, accuracy, and precision of newer methods of CO measurement generally involve a comparison with more established techniques, such as TD. In the past, correlation and regression analysis was used, however, Bland-Altman analysis has become the preferred method of statistical analysis for determining level of agreement. The difference between comparative measurement is plotted (the bias) against the mean values of each pair of readings. The standard deviation (SD) of each bias measurement is calculated and 95% confidence limits drawn (*μ* ± 2SD). The latter is the limits of agreement, upon which a determination of precision is based [33].

L. A. H. Critchley and J. A. J. H. Critchley [33] performed a meta-analysis and found wide variations in the presentation of statistical data for comparison studies. They advocated that studies present the mean CO (*μ*), the bias, the limits of agreement (95% C.I.), and the percentage error (±2SD/*μ*) and concluded that acceptance of a new technique should rely on limits of agreement of up to ±30%. They point out that the Bland-Altman method does not compensate for the magnitude of the measurements and the size of the error and suggest that percentage error be calculated for each set of data as opposed to calculating it one time from the averaged data.

## 7. Pulse Power Analysis

Pulse power analysis is based on the theory that fluctuations of blood pressure about the mean are directly related to the stroke volume (SV) ejected into the arterial system [34]. Accuracy of measurement is complicated by several factors.

- Nonlinear compliance of the arterial wall. Decreased aortic compliance occurs at higher than at lower blood pressures (BPs).
- Wave reflection, since pulse pressure detected in a peripheral artery is a composite of the pressure wave from ejection from the heart and the reflected pressure wave from the distal arterial tree. Changes in systemic vascular resistance (SVR) affect the reflected wave augmentation of the arterial pressure. The size of the reflected waves is also found to vary with the sampling site proximity to the central circulation and patient age.
- Damping of the transducer system.
- Aortic systolic outflow. Filling is pulsatile; however, outflow tends to be continuous [34].

The LiDCO method of pulse power analysis utilizes a proprietary autocorrelation algorithm (PulseCO (LiDCO, Cambridge, UK)) which addresses the factors mentioned above. The assumption made is that, following calibration and correction for compliance, there is conservation of mass/power and so a linear relationship exists between net power and net flow. Autocorrelation allows for the determination of the beat period as well as the net power change across the whole beat from the stroke volume. As a result, the effect of reflected waves is negated. Since the method is time based, the effects of arterial damping are minimized. Apart from extreme conditions, the pulse power tends to remain the same regardless of the degree of damping [34]. 

The LiDCO plus (Cambridge, UK) system is coupled to a lithium dilution system, a technique first described by Linton et al. [35] in 1993. Either central or peripheral venous access may be used in addition to a peripheral arterial line, to which a disposable lithium sensitive sensor is attached. The sensor membrane contains an ionophore which is selectively permeable to lithium [36]. The membrane voltage is related to the plasma lithium concentration using the Nernst equation. The voltage is amplified and digitalized for analysis. Sodium supplies the baseline voltage in the absence of lithium. 

The LiDCO plus monitor requires CO calibration with lithium dilution once every eight hours according to the manufacturer. It has been suggested, however, based on recent data, that repeat calibration should take place during major hemodynamic changes [37]. Cecconi et al. [38] concluded that, for good precision, three lithium dilution measurements should be performed. During calibration, isotonic lithium chloride (150 mM) is given intravenously (0.02 to 0.04 mmol/kg). CO is derived from the dose and the area under the concentration-time curve. Since lithium is only distributed in the plasma fraction of blood, for the determination of CO, blood flow is determined by dividing plasma flow by 1-packed cell volume, assessed on the basis of hemoglobin/33 [2]. 

The accuracy of the Pulse CO algorithm may be compromised under the following circumstances:

- aortic valve regurgitation,
- post-aortic reconstruction,
- intra-aortic balloon pump,
- highly damped peripheral arterial lines,
- severe peripheral arterial vasoconstriction,
- inaccurate sodium and hemoglobin measurements,
- arrhythmias,
- intra- or extracardiac shunts.

In addition, each 1 g/dL difference in hemoglobin artifactually results in a 4% change in the CO measurement [39]. 

Lithium therapy is a contraindication to the use of LiDCO, as overestimation of CO occurs with elevated background levels. Some nondepolarizing muscle relaxants contain high levels of quaternary ammonium residues, causing the electrode to drift. Recalibration is recommended prior to injection of the drugs or after the peak concentration has fallen. Bolus dosing is also recommended if nondepolarizing muscle relaxant use cannot be avoided.

The safety of lithium use has been well established. Since lithium does not occur naturally in plasma, does not bind to plasma or tissue proteins, and is not lost in passage through the pulmonary circulation, tiny doses may be used. Levels achieved are <1% of therapeutic levels used during treatment of mania with lithium carbonate [36].

For the newer LiDCO rapid (Cambridge, UK), lithium dilution has been replaced with a nomogram which has been derived from in vivo data, to estimate CO. The system features simplicity and ease of use. It was designed to provide reliable hemodynamic trends, which would be useful for goal-directed fluid therapy. In a clinical setting where absolute values for SV and SVR are required, a calibrated, system is warranted [40].

### 7.1. Validation Studies

Investigators studying small patient populations under different clinical settings and with different reference standards have reported variable findings regarding the accuracy of the LiDCO system. While some have suggested acceptable accuracy [41–44], others have found unacceptable accuracy compared to PAC-derived CO [45, 46]. 

Linton et al. [35] studied 9 patients immediately after-cardiac-surgery and reported good correlation (*r* = 0.89) and a bias 0.3 (0.5) L/min between LiDCO and intermittent bolus pulmonary artery TD (PATD). Costa et al. [47] reported agreement between LiDCO and intermittent PATD in 23 after-liver-transplant patients exhibiting the typical hyperdynamic circulation; the reported bias and 95% limit of agreement for the PAC versus PulseCO_Li_ were 0.29 L/min (*r* = 0.85) with a percentage error of 16.8%. 

The validity of the device has also been studied in pediatric patients. Kim et al. [48] reported good correlation (*r* = 0.94) with PATD in 20 children (age range 2.5–15.5 years) undergoing cardiac catheterization. In smaller children (<20 kg), a separate analysis still showed good correlation (0.89). Linton et al. [49] compared the device against transpulmonary TD in 20 pediatric patients (age range 5 days–9 years) and also reported good accuracy (*r* = 0.96).

Yamashita et al. [46] compared bolus PATD with the PulseCO, calibrated with CO by the TD method in patients during cardiac-surgery. They found poor correlation (*r* = 0.49–0.55) and large bias (0.3–0.76), concluding that the methods were not interchangeable. In an observational study of 8 after-cardiac-surgery patients, McCoy et al. [45] compared continuous cardiac index monitoring (CCI) with LiDCO, using a peripheral iv line for indicator delivery. The investigators found minimal bias (−0.01) but wide 95% limits of agreement with respect to the mean, suggestive of clinically significant differences.

In a randomized controlled trial, Pearse et al. [50] used LiDCO to guide goal-directed therapy (GDT) in high-risk surgical patients. The outcome was fewer complications and shorter length of hospital stay in the GDT group. 

Costa et al. [51] carried out comparisons between the LiDCO rapid and intermittent and continuous PATD in 10 after-liver-transplant patients. Their preliminary data showed that, the LiDCO rapid provided acceptable readings, with percentage errors of 26 and 30%, respectively, compared with intermittent and continuous PATD. 

Multiple studies are ongoing using the LiDCO rapid to gauge fluid responsiveness and guide fluid management. [52–54]. The LiDCO rapid is also currently being used in a large government-supported multicenter trial currently underway in the UK, OPTIMISE, aimed at improving surgical outcomes by optimizing cardiovascular management [55].

## 8. Pulse Contour Analysis

Pulse contour analysis for CO measurement is based on the hypothesis that the area under the curve of the systolic part of the arterial pressure waveform is proportional to the SV [56]. Wesseling et al. [57] developed the first successful algorithms in the 1970's, which continuously analyze the pressure waveform from an arterial line. The area under the systolic portion of the arterial pulse wave (measured from the end of diastole to the end of the ejection phase) divided by the aortic impedance gives a measure of the stroke volume, which multiplied by the heart rate gives the cardiac output.

### 8.1. The PiCCO System

The PiCCO system (PULSION Medical Systems) is the first pulse contour device to be introduced into clinical practice [4, 56]. In 2007, the PiCCO2 replaced the PiCCO. 

External manual calibration of the system is performed via transpulmonary TD every eight hours, or up to hourly during periods of hemodynamic instability [58, 59]. Blood temperature changes from a thermo-indicator solution injected via a CVC are detected by a thermistor-tipped catheter, typically placed in the femoral artery. Alternatively, the radial, axillary, or brachial artery may be used; however, longer catheters are required to adequately assess the aortic pressure wave signal from more distal sites. Although accuracy of the transpulmonary TD technique may be affected by the longer transit time, errors due to airway pressure variation are eliminated. The calibration is repeated three to five times to obtain a calibration factor for calculation of continuous CO, intrathoracic blood volume (ITBV), and extravascular lung water (EVLW).

Global end-diastolic volume (GEDV) is also measured and, together with ITBV, is representative of cardiac preload and EVLW. EVLW, comprising intracellular, interstitial, and intra-alveolar water, is measured intermittently using transpulmonary TD as a means of quantifying pulmonary edema [60]. Systolic pressure variation and stroke volume variation (SVV) provide information about volume status in mechanically ventilated patients [32].

### 8.2. Limitations

The accuracy of analysis is influenced by vascular compliance, aortic impedance, and peripheral arterial resistance. Second generations of the system software address issues related to differences in individual patients' aortic compliance and now analyze the shape of the waveform and the pulsatile systolic area [61].

Results may be altered secondary to technical problems such as air bubbles in the system, clotting of the catheter, and inadequate indicator. Problems with analysis are also seen with severe arrhythmias, raised EVLW (requiring more indicator), aortic aneurysm (causes ITBV and GEDV to be overestimated), severe valve insufficiency (CO is correct, but preload is overestimated), and rapidly changing body temperature. Recirculation of thermo-indicator may occur in patients with intracardiac shunts and in pediatric patients with open ductus Botalli [60].

### 8.3. Validation Studies

The pulse contour analysis method has largely been found to correlate well against pulmonary artery thermodilution (PATD) in numerous studies under varying conditions, including coronary artery bypass grafting (CABG) [62–65]. Some bias should be recognized, however, since TD is required for calibration [66]. Other investigators have reported large discrepancies between the two techniques. Halvorsen et al. [67] reported unacceptably large discrepancies with the PATD during off-pump CABG. For lung transplantation, good correlation was found between PiCCO and TD [68]. Significant errors during periods of hemodynamic instability, with the need for repeated recalibration has been reported [69]. In burn patients, good correlation during low to normal CO was reported; however, greater discrepancy was seen at high cardiac indices [70].

## 9. FloTrac/Vigileo

The FloTrac Vigileo (Edwards Lifesciences, LLC, Irvine Calif, USA) is another pulse contour device, which was first introduced in April, 2005 [4]. The device provides continuous CO measurement from a proprietary FloTrac sensor attached to a standard peripheral arterial catheter, which is connected to the Vigileo monitor. Calculations of SVR and SVV are also displayed. A significant feature of the system is that, unlike PiCCO and Pulse CO, external calibration is not required as the algorithm performs its own calibration using patient demographics and waveform analysis [4]. Notably, no central venous line is required.

The FloTrac algorithm integrates multiple characteristics of the arterial pressure waveform with patient specific demographic data. Parameters include heart rate (HR), the standard deviation of the arterial pressure, a scale factor proportional to vascular and peripheral resistance combined over the arterial pressure waveform (mean, standard deviation, skewness and kurtosis), pressure-dependent Windkessel, compliance and body surface area [71]. The standard deviation of pulse pressures sampled over 20 seconds is correlated with a predicted SV based on demographic data (age, gender, height, and weight) and extrasystoles and other minor artifacts are eliminated via a beat-detection algorithm [37]. Impedance is also determined from the demographic data. Vascular compliance and resistance are derived from arterial waveform analysis [56]. 

Second generation versions (1.07 or later) undergo calibration every minute, with improved CO measurement compared with earlier versions [37]. The third generation device, with its Dynamic Tone Technology, is purported to use additional physiologic variables, with automatic adjustment for changes in vascular tone [72]. The third generation is undergoing investigations during hemodynamic instability, such as sepsis and acute circulatory failure [73]. When used in conjunction with a central venous pressure catheter, systemic vascular resistance (SVR) and systemic vascular resistance index (SVRI) may be calculated [71].

### 9.1. Limitations

Since the calculations depend on the fidelity of the waveform, good arterial signal quality is critical to accuracy of CO measurement. Unreliable measurements are seen in the presence of arrhythmias and during intra-aortic balloon pump use. Although any arterial site may be cannulated, Compton et al. [74] found errors introduced when different sites had mean arterial pressure (MAP) differences of 5 mmHg or more. Mayer et al. [75] reported the percentage error for the FloTrac/Vigileo in obese patients, with their altered arterial compliance, to be slightly higher than that in nonobese when compared with pulmonary artery TD.

### 9.2. Validation Studies

Although some investigators have reported that the FloTrac appears to be reliable in several situations, its reliability is questioned in hemodynamically unstable patients [74, 76]. Manecke and Auger [77] found satisfactory correlation with PATD for clinical use in after-cardiac-surgery patients, and in a multicenter trial, McGee et al. reported the FloTrac to be comparable to PATD in critically ill patients [78]. 

Biancofiore et al. [79] found limited accuracy in patients with low SVR who were undergoing liver surgery. Similarly, Matthieu et al. [80] and Krejci et al. [81] found poor agreement in liver-transplant patients with low SVR compared with PATD. Hamm et al. [71] compared the device with instantaneous readings from a pulmonary artery catheter in nine patients undergoing CABG and concluded that the two were not clinically equivalent. Sakka et al. concluded that transpulmonary TD was more accurate than with FloTrac in septic patients [82]. 

The manufacturer reports that the system's third generation algorithm (software version 3.02) has broadened its database to include more patients with hyperdynamic conditions and is undergoing investigation [72]. Mayer et al. [83] found only moderate correlation with PATD (overall percentage error of 46%) in patients undergoing CABG with software version 1.03; however, they later reported percentage errors of 28.3% and 20.7% intraoperatively and ICU, respectively, when studying the 1.1 software version in a similar patient population [84]. 

Further studies are warranted to validate the device's reliability under varying physiologic states. Hofer et al. [85] compared the device with the PiCCO to determine how well fluid responsiveness could be predicted using SVV and found similar accuracy. Suehiro and Okutani [86] concluded that SVV as measured by the FloTrac system was able to predict fluid responsiveness in patients on one lung ventilation. High risk patients undergoing major abdominal surgery who received goal-directed fluid therapy using the FloTrac/Vigileo device (software version 1.14) were found to have fewer complications and decreased hospital LOS [87].

## 10. The NICO System: Fick's Principle Using Carbon Dioxide

The NICO system (Novametrix Medical Systems, Wallingford, Conn, USA), first introduced in 1999 [4], uses the differential Fick partial rebreathing technique to measure CO in intubated, sedated, mechanically ventilated patients. 

Fick's principle, using CO_2_ as an indicator, is rewritten as follows:

(4) $$\text{CO}=\frac{{\text{VCO}}_{2}}{{\text{Cv}}_{\text{C}{\text{O}}_{\text{2}}}-{\text{Ca}}_{\text{C}{\text{O}}_{\text{2}}}},$$

where VCO_2_ is elimination of Ca_CO_2__ and Cv_CO_2__ is arterial and venous CO_2_ content, respectively. 

Ca_CO_2__ may be calculated from the PaCO_2_ or estimated from the end-tidal CO_2_. Diffusion abnormalities limit the accuracy of estimation [32]. VCO_2_ is calculated from the difference between inspired and expired CO_2_ content. Cv_CO_2__ is estimated by using a partial rebreathing technique.

A proprietary disposable rebreathing loop is attached to the ventilator circuit, in addition to a mainstream infrared CO_2_ sensor, a fixed orifice differential pressure pneumotachometer, and a rebreathing valve. Every three minutes, partial rebreathing is initiated by opening the rebreathing valve, which adds 150 mL of dead space to the circuit. The difference between normal and rebreathing ratios are used to calculate pulmonary blood flow [4, 88]. Shunt correction is carried out using Nunn's isoshunt curves, a series of curves that describe the relationship between PaO_2_ and FiO_2_ for different levels of intrapulmonary shunt. Shunt is determined by using the PaO_2_ and FiO_2_. 

The intubated patients must be able to tolerate the brief period of rebreathing. Ventilator settings may need adjustment due to the at least 35 mL of increased dead space introduced.

### 10.1. Limitations

The normal difference between mixed venous and arterial CO_2_ tension is approximately 6 mmHg. Any increase, due, for example, to increased dead space, would lead to changes in the calculated CO too. The PaCO_2_ and PvCO_2_ relationship is only valid when the PaCO_2_ is more than 30 mmHg and when the CO_2_-Hgb dissociation curve is linear. Hyperventilation to a PaCO_2_ < 30 mmHg would lead to inaccuracies in CO measurement. Since only nonshunted blood is measured, the shunt fraction must be estimated for an accurate measure of CO. The shunt fraction is estimated using the shunt equation:

(5) $$\frac{Q_{s}}{Q_{\text{T}}}=\frac{{\text{CcO}}_{2}-{\text{CaO}}_{2}}{{\text{Cc}}_{{\text{O}}_{\text{2}}}-{\text{CvO}}_{2}}$$

where CaO_2_, CvO_2_, and CaO_2_ are the end-capillary, venous, and arterial oxygen content. To measure these noninvasively, Nunn's isoshunt plots are used.

### 10.2. Validation Studies

Variable results have been published using this technique, with many studies involving patients with varied degrees of intrapulmonary shunt in settings from cardiac-surgery or in hemodynamically unstable ICU patients [32]. Moderate agreement during thoracic surgery was found compared with pulmonary artery TD [89].

Kotake et al. [90] found improved correlation with TD with newer software versions compared with previous studies in patients undergoing abdominal aortic aneurysm repair [91]; however, they concluded that the technology still has not reached the level of interchangeability. In a small study of patients undergoing hip replacement, NICO was compared to TD and a slight underestimation was found, with a small degree of bias. For off-pump CABG patients, investigators concluded that NICO reliably and more rapidly measured CO compared with TD. The authors reported the tendency to underestimation perioperatively, but overestimation in the postoperative period. The limits of agreement were reported to be larger intraoperatively than postoperatively [92]. Similar values were obtained from NICO and PATD for patients before undergoing cardiopulmonary bypass (CPB); however, after separation, NICO tended to underestimate CO [93]. Other investigators comparing NICO and TD for major surgery or the ICU found that NICO slightly underestimated CO compared to TD [94]. Following CABG, NICO had insufficient agreement with TD, as opposed to pulse contour [95]. NICO was found to underestimate CO compared with TD in ICU patients after cardiac-surgery and found least suitable where CO was high [96]. Poor agreement was reported in a similar patient group [97]. Decreased correlation has been reported in the setting of high CO, decreased minute ventilation, increased intrapulmonary shunt, or severe chest trauma [32]. Rocco et al. [98] reported bias of −2.3 L/min when *Q*_*s*_/*Q*_*p*_ exceeded 35%.

## 11. Thoracic Bioimpedance

Thoracic bioimpedance (TEB) is the least invasive of the CO monitors. The technology was first developed by Kubicek et al. [99] in the 1960's, with the initial testing being carried out on astronauts [100]. The basis for its use was later pioneered by Lababidi et al. [101] in 1970, with subsequent improvements carried out over the following decades, based on animal and human research. The technique finally became popularized based on studies by Shoemaker et al. in the 1990's [100, 102].

The underlying theory is that the thorax is a cylinder perfused with fluid (blood) which has a specific resistivity. Bioimpedance is the electrical resistance to a high-frequency low-amplitude current transmitted from electrodes placed on the upper and lower thorax [32]. Typically, six electrodes are placed—two on either side of the neck and four in the lower thorax. Current transmitted from the outermost surface electrodes is sensed by the innermost set of surface electrodes. The impedance (*Z*_*o*_) is calculated from the voltage changes, which are indirectly proportional to the volume of fluid in the thorax, such that increased fluid results in lower TEB [103]. Blood flow from the aorta is primarily responsible for the change in impedance. Stroke volume is estimated based on the formula

(6) $$\text{SV}=\frac{\rho \left(L^{2}\right)}{\left(Z_{\Phi }^{2}\right)}\cdot \left[{\text{VET}}_{x}\frac{\left(d_{z}\right)}{\left(dt_{\max\;}\right)}\right],$$

where *ρ* is the resistivity of blood (ohm-cm), *L* is the distance between electrodes (cm), *Z*_Φ_ is the mean thoracic impedance between electrodes (ohm), VET is the ventricular ejection time (sec), and (*dz*/*dt*)_max_ is the maximum negative slope of the bioimpedance signal (ohm/sec) [89]. Several hemodynamic parameters may be calculated using HR and noninvasive blood pressure, together with the SV [103].

### 11.1. Limitations

TEB is affected by a number of factors [32]:

- changes in tissue fluid volume,
- respiration-induced changes in the volume of pulmonary and venous (“noise” must be filtered out from the desired changes in volumetric blood flow of the aorta),
- changes in electrode contact or position,
- arrhythmias—the VET is determined using the interval between QRS complexes,
- acute changes in tissue water, for example, pulmonary or chest wall edema or pleural effusions,
- noise from electrocautery, mechanical ventilation and surgical manipulation,
- changes in myocardial contractility, for example, from anesthetic drugs or ischemia.

### 11.2. Validation Studies

Several investigators found that TEB compared favorably with PATD in varying settings including during cardiac catheterization, surgical patients, and emergency room patients [102, 104–108]. Van De Water et al. [109] found the TEB compared favorably with TD in post cardiac surgical patients. Kööbi et al. [110], using whole-body impedance cardiography in CABG patients, reported excellent repeatability which allowed for continuous monitoring. Spiess et al. [111] used BioZ (SonoSite Inc, Bothell, Wash,USA) intraoperatively for patients undergoing CABG and found that the technique initially compared well with TD, but, immediately postoperatively, the Bland-Altman analysis was not as robust. Of note, good correlation was seen during opening of the chest. Spinale et al. [112] used TEB for post-CABG patients and found good correlation with TD but poor correlation in patients who developed severe tachycardia and frequent arrhythmias.

Several investigators have found poor reliability and poor correlation with PATD in after-cardiac-surgery, the critically ill and the elderly [113–115]. In a meta-analysis performed by Rotcajg et al. [116], the conclusion was that TEB might be useful for trend analysis but not diagnostic interpretation. Correlation appeared to be better with repeated measurement designs. Atherosclerotic changes in the aorta of elderly patients reduces the Windkessel effect and contributes to increased inaccuracy [114]. 

TEB appears unlikely to become a routine monitor of CO for anesthesia or critical care unless further refinements in signal processing occur.

## 12. Thoracic Bioreactance

Thoracic bioreactance technology developed as a refinement of TEB. Bioreactance analyses beat-to-beat changes in the phase of electrical voltage signal relative to the applied current signal across the thorax. Changes in intrathoracic volume produce variations in electrical capacitive and inductive properties (bioreactance). The techniques for detecting relative phase shifts are powerful and less affected by noise and external interference [115]. Thoracic bioreactance technology is commercially available as the NICOM system (Cheetah Medical Inc., Indianapolis, Ind, USA). Two dual-electrode stickers are placed on either side of the thorax—one electrode is used to inject the sine-wave high-frequency (75 kHz) current into the body and the other is used by the voltage input amplifier [115]. The final measurement is determined by averaging the 2 signals.

### 12.1. Validation Studies

Several validation studies of thoracic bioreactance have been conducted, using continuous PATD as the reference continuous technique. Investigators report good correlation between the two methods (*r* = 0.64–0.9) and minimal bias [115–117]. Comparisons are limited by differences in intrinsic variability of measurements of PATD and differences in the time responsiveness of the 2 modalities. In addition, PATD only measures right ventricular output, excluding the bronchial circulation. For this reason, Rotcajg et al. [116] considered 20% bias and precision as acceptable. 

Smaller studies comparing NICOM with PICCO and Vigileo devices report similar capabilities between the devices [116, 118].

### 12.2. Limitations

The assumption that the area under the flow pulse is proportional to the product of peak flow and VET may not be valid under periods of low flow, and readings may have decreased accuracy [115].

## 13. Endotracheal Cardiac Output Monitor

The endotracheal cardiac output monitor (ECOM; ConMed, Irvine, Calif, USA) measures CO using impedance plethysmography. The ascending aorta lies in close proximity to the trachea. Using the principle of bioimpedance, a low frequency current of 2 mA and 200 kHz is delivered from electrodes attached to a standard endotracheal tube (ETT) [119].

The ECOM 6 3D endotracheal tube (ETT) is a standard ETT to which are attached three orthogonal pairs of sensing electrodes on the cuff. Current is delivered between an electrode on the shaft of ETT and the number three electrode on the balloon. The sensing electrodes on the cuff detect the change in impedance secondary to aortic blood flow. The three-dimensional array allows for up to twelve combinations of electrodes which may be used for measurement of flow. This compensates for positional and anatomical differences between the cuff and aorta [119].

A proprietary algorithm calculates SV based on impedance changes. Increased blood flow in the aorta leads to decreased impedance. Apart from CO, also displayed are HR, ECG waveforms, SV, CI, and SVR [120].

### 13.1. Limitations

Coronary blood flow, which represents about 4-5% of CO is not recorded. Electrocautery produces interference.

### 13.2. Validation Studies

The technology is not yet fully validated in humans. A porcine study found excellent correlation when compared with transit time flow probes [120]. A study in cardiac-surgery patients reported poor correlation with TD, wide limits of agreement and a large percentage error [121].

## 14. Ultrasound Dilution

Ultrasound dilution (UD) is a minimally invasive technique, first introduced in 1995, and widely used in hemodialysis and in extracorporeal membrane oxygenation (ECMO) to measure shunt flow, vascular access recirculation and CO [122]. The technique uses isotonic saline as an indicator to measure hemodynamic variables. 

The underlying principle is that blood ultrasound velocity is a function of total blood protein concentration, temperature and plasma average ion concentration. The injection of isotonic saline results in decreased blood ultrasound velocity, from which dilution curves can be produced [123]. The setup involves a disposable tubing, which is used to create an extracorporeal loop between existing peripheral arterial and central venous catheters. The arteriovenous loop is primed with heparinized saline. A roller pump circulates blood from the artery to the vein. Two reusable sensors are clamped onto the arterial venous limbs of the loop. These sensors measure the changes in blood ultrasound velocity and blood flow following a bolus of saline injected into the venous side. The CO calculation is based on the Stewart-Hamilton principle [123].

### 14.1. Validation Studies

Relatively few studies investigating the technology have been undertaken thus far. Galstyan et al. [122] compared CO and blood volumes using UD and PiCCO technology in adult ICU patients and concluded that the two were equivalent and interchangeable in that patient population. PiCCO blood volumes were significantly higher. The technology appears to be able to be used in different patient population groups. Krivitski et al. [123] performed in vitro studies to confirm the ability of UD technology to measure small flows and volumes in pediatric patients and neonates. Tsutsui et al. [124] found good correlation with TD in patients undergoing abdominal surgery.

## 15. Transesophageal Echocardiography

Transesophageal echocardiography (TEE) is widely used in the perioperative setting for evaluating cardiac anatomy and function. Doppler techniques for the measurement of CO are most commonly based on Simpson's rule. Early operators made determinations using the pulmonary artery, which only reflects right ventricular CO. Two-dimensional echocardiography determined the cross-sectional area of the PA, which was multiplied by the integral of the instantaneous flow velocity, determined by pulsed wave Doppler in the plane of the cross section [125]. A drawback is difficulty visualizing the PA in a significant number of patients because it may be obscured by the left main stem bronchus.

The validated frequently used technique developed by Perrino et al. [126] determines the cross-sectional area of the left ventricular outflow tract (CSA_LVOT_) in a mid-esophageal aortic long axis view. Planimetry is used to measure the area of the aortic valve. To measure aortic blood flow, the probe is positioned in a transgastric short-axis view of the left ventricle at the mid-papillary level. The image array is rotated approximately 120° to produce imaging of the LVOT and ascending aorta lying parallel to the ultrasound beam. Continuous-wave Doppler is used to measure aortic blood flow velocities at the level of the aortic valve. Doppler CO is calculated as a product of the velocity time integral, CSA_LVOT_, and HR [127]. Another method described uses the transgastric, apical view to assess aortic blood flow. The ultrasound beam is oriented almost parallel to the aortic valve blood flow. Probe positioning for this view is technically challenging [61].

## 16. Esophageal Doppler

Esophageal Doppler (ED) utilizes a flexible probe, approximately the size of a nasogastric tube, at the tip of which is a transducer (4 MHz continuous or 5 MHz pulsed wave). The probe may be left in place for days to weeks in intubated, sedated, mechanically ventilated patients. When advanced to the mid-thoracic level, ideally between the 5th and 6th thoracic vertebrae, the device is parallel to and thus able to measure blood flow velocity in the descending aorta [128]. It is assumed that the aorta is a cylinder and flow is calculated by multiplying the cross-sectional area (CS_a_) by the velocity (*V*_*f*_). Since velocity changes with pulsatility of flow, *V*_*f*_ is described as the area under the curve of a velocity-time graph [32]. The area is calculated as the integral of the velocity curve over time (*dV*/*dt*) from the start to the end of aortic blood flow (*T*_0_ and *T*_1_, resp.). This area, known as the stroke distance, is the distance travelled by the blood during systole (cm). This value is multiplied by the CSA_a_. The aortic area may be derived either from published nomograms or direct measurement [32]:

(7) CO=HR×SV.

SV changes can be used to guide fluid administration. ED also has the capability of determining the corrected time flow (FTc), which is the systolic flow time corrected for an HR of 60/min. This value, which represents the time from the beginning of the aortic waveform upstroke to its return to baseline, is used as a measure of cardiac preload. Good correlation with other techniques, such as pulmonary artery occlusion pressure, along with improved outcomes has been reported [129–132].

### 16.1. Limitations

Only descending aortic blood flow, which represents about 70% of total flow, is measured, and so a correction factor (*K*-factor) must be added to compensate for the blood flow to aortic arch vessels. This flow ratio may vary with metabolic activity, between different organs and during hemodynamic instability, and the validity is questionable outside of young healthy patients [32]. An inconstant proportion of blood flow to the descending aorta may also occur in the setting of aortic coarctation, aortic cross clamp, and pregnancy. Turbulence due to thoracic aneurysms, aortic balloon pump, aortic valve disease interfere with the validity of results [133]. 

CSA_a_ changes with variations in pulse pressure, vascular tone, aortic compliance, volume status, or catecholamine use. Direct measurement produces greater accuracy [32]. In 76 patients with acute circulatory failure, measurements of aortic blood flow before and after a fluid bolus revealed an underestimation of the response to the fluid bolus in a significant number of patients if readings were based on an estimated unchanged CSA_a_ as opposed to directly measured aortic velocity and CSA_a_ [134]. Proper probe position is essential to accuracy of determination of *V*_*f*_. The Doppler beam must be within 20° of axial flow to obtain good measurement.

With respect to fluid management, interpretation of FTc may be complicated by its inverse relationship with SVR. In conditions of elevated SVR, such as heart failure or excessive vasopressor use, FTc is reduced and may prompt fluid administration. Other conditions, such as pericardial tamponade or mitral stenosis, where there is limited cardiac filling will produce a decreased FTc and again prompt further fluid administration in a scenario where the patient may already have optimal cardiac filling based on the starling curve. SV has thus been argued to be a preferable variable to monitor fluid status [135, 136].

### 16.2. Validation Studies

Multiple studies have compared the validity of ED for measurement of CO against PATD under varying conditions [129, 137–139]. Dark and Singer [139] published a meta-analysis of eleven studies in critically ill patients, finding a pooled median bias of 0.19 L/min (range: −0.69–2.0 L/min) for CO. Boulnois and Pechoux [140] reported the pooled limits of agreement for 3 studies including 90 patients under a range of flow states to be −2.21 to 2.33 L/min. Laupland and Bands [133], in a meta-analysis of 25 studies, concluded that ED was reliable, responsive to changes, was showed good agreement with low bias, however, the wide limits of agreement raised concerns about precision. The two techniques are therefore not thought to be interchangeable; however, ED may be used to track changes [137].

Improved patient outcome has been demonstrated by a number of investigators when ED is used in goal-directed fluid therapy. Sinclair et al. [132] reported ED-guided fluid loading resulted in greater improvements in SV and CO with fluid administration in study patients, as well as faster recovery and decreased LOS than in controls. Venn et al. [141] similarly reported reduced hypotension and faster recovery for ED-monitored patients undergoing femoral fracture repair, compared with controls who received central venous pressure monitoring. In patients having major elective surgery, Gan et al. [142] reported earlier return to bowel function and decreased incidence of postoperative nausea and vomiting. Mythen and Webb [131] found a decreased incidence of gut mucosal perfusion (measured by gastric tonometry), major complications, and decreased hospital and ICU stay in cardiac-surgery patients who received goal-directed colloid therapy guided by ED compared with standard management. Wakeling et al. [143] randomized 128 patients receiving colorectal surgery to fluid management with guided with ED or central venous pressure monitoring. Decreased hospital LOS and faster gut recovery were seen in the ED-guided group. Noblett et al. [144] reported shorter hospital stay and decreased morbidity in patients undergoing colorectal resection who received ED-guided fluid management. Additionally, the intervention group had lower levels of interleukin 6, which may be a reflection of improved bowel perfusion. Conway et al. [145] reported improved hemodynamics in patients having major bowel surgery and fewer ICU admissions. In trauma patients, ED-guided fluid therapy resulted in decreased blood lactate levels, reduced infectious complications and decreased hospital and ICU LOS [146]. A nurse delivered ED-guided fluid protocol in patients after-cardiac-surgery resulted in shortened hospital LOS [147].

## 17. Conclusion

Despite controversies, complications, and inherent errors in measurement, intermittent bolus PATD CO measurement continues to be the gold standard. Newer techniques provide less invasive alternatives and will be increasingly adopted over time; however, the currently available monitors are still unable to provide central circulation pressures or true mixed venous saturations and cannot replace the PAC [32]. Many minimally invasive techniques continue to suffer from decreased accuracy and reliability under periods of hemodynamic instability, and so few have reached the level of interchangeability with the PAC. Esophageal Doppler and pulse contour monitors have the advantage of being able to predict fluid responsiveness. Their use in GDT has already been shown to decrease postoperative morbidity, and the use of these technologies is anticipated to continue to lead to greater improvement in outcomes [32].

## Figures and Tables

**Table 1:** Comparison of minimally invasive cardiac output monitoring techniques (CI: cardiac index, HR: heart rate, and ECG: electrocardiogram).

Technique	Advantages	Additional variables	Invasiveness	Limitations
LiDCO plus	Continuous CO measurement	SV	Arterial line	Requires good fidelity of arterial waveform
	Useful in goal-directed therapy	SVV	Peripheral or central venous line	Calibration affected by neuromuscular blockers
				Contraindicated in lithium therapy
				Requires transpulmonary lithium dilution calibration
PiCCO plus	Continuous CO measurement	GEDV	Arterial line	Requires good fidelity of arterial waveform
		EVLV		Requires transpulmonary thermodilution calibration
		SVV		
		PPV		
				
FloTrac/Vigileo	Continuous CO measurement	SVV	Arterial line	Requires good fidelity of arterial waveform
	No calibration required			
NICO	Ease of use	Shunt	Endotracheal intubation	Affected by changes in dead space or V/Q matching
		Ventilatory variables	Valid only with PaCO_2_ > 30 mmHg	
				
Bioimpedance	Noninvasive		Cutaneous electrodes	Affected by electrical noise, movement
	Continuous CO measurement			Electrode contact affected by temperature and humidity
				Requires hemodynamic stability
				Not useful in dysrhythmias
Bioreactance	Noninvasive Continuous CO measurement		Cutaneous electrodes	
ECOM		SV	Endotracheal intubation	Coronary blood flow not recorded
		CI		Electrocautery produces interference
		SVR		No fully validated human studies
		HR, ECG		
Ultrasound dilution	Measures flow in ECMO and hemodialysis circuits		Arterial line	Fluid overload with saline injection in sensitive patients
			Central venous catheterization	Errors from indicator loss in inadequate lung perfusion
				Errors in the presence of septal defects
TEE	Used to evaluate cardiac	SV	Esophageal probe	Mainly used perioperatively
	anatomy and function, preload, and myocardial ischemia			
Esophageal Doppler	Useful in goal-directed therapy	SV	Esophageal probe	Measures only descending aortic flow
				Assumptions about aortic size may be erroneous

## References

[1] Prys-Roberts C (1969). The measurement of cardiac output. *British Journal of Anaesthesia*.

[2] Reuter DA, Huang C, Edrich T, Shernan SK, Eltzschig HK (2010). Cardiac output monitoring using indicator-dilution techniques: basics, limits, and perspectives. *Anesthesia and Analgesia*.

[3] Kadota LT (1985). Theory and application of thermodilution cardiac output measurement: a review. *Heart and Lung*.

[4] Stewart GN (1921). The output of heart in dogs. *American Journal of Physiology*.

[5] Stewart GN (1897). Researches on the Circulation Time and on the Influences which affect it. *The Journal of Physiology*.

[6] Nishikawa T, Dohi S (1993). Errors in the measurement of cardiac output by thermodilution. *Canadian Journal of Anaesthesia*.

[7] Swan HJ, Ganz W, Forrester J, Marcus H, Diamond G, Chonette D (1970). Catheterization of the heart in man with use of a flow-directed balloon-tipped catheter. *New England Journal of Medicine*.

[8] Libby P, Braunwald E (2007). *Braunwald’s Heart Disease*.

[9] Jansen JRC (1995). The thermodilution method for the clinical assessment of cardiac output. *Intensive Care Medicine*.

[10] Nishikawa T, Dohi S (1993). Errors in the measurement of cardiac output by thermodilution. *Canadian Journal of Anaesthesia*.

[11] Ganz W, Donoso R, Marcus HS, Forrester JS, Swan HJC (1971). A new technique for measurement of cardiac output by thermodilution in man. *The American Journal of Cardiology*.

[12] Stetz CW, Miller RG, Kelly GE, Raffin TA (1982). Reliability of the thermodilution method in the determination of cardiac output in clinical practice. *American Review of Respiratory Disease*.

[13] Elkayam U, Berkley R, Azen S (1983). Cardiac output by thermodilution technique. Effect of injectate's volume and temperature on accuracy and reproducibility in the critically ill patient. *Chest*.

[14] Bazaral MG, Petre J, Novoa R (1992). Errors in thermodilution cardiac output measurements caused by rapid pulmonary artery temperature decreases after cardiopulmonary bypass. *Anesthesiology*.

[15] Groeneveld ABJ, Berendsen RR, Schneider AJ, Pneumatikos IA, Stokkel LA, Thijs LG (2000). Effect of the mechanical ventilatory cycle on thermodilution right ventricular volumes and cardiac output. *Journal of Applied Physiology*.

[16] Miller RD (2004). *Miller's Anesthesia: 2-Volume Set*.

[17] Leibowitz AB, Oropello JM (2007). The pulmonary artery catheter in anesthesia practice in 2007: an historical overview with emphasis on the past 6 years. *Seminars in Cardiothoracic and Vascular Anesthesia*.

[18] Gore JM, Goldberg RJ, Spodick DH, Alpert JS, Dalen JE (1987). A community-wide assessment of the use of pulmonary artery catheters in patients with acute myocardial infarction. *Chest*.

[19] Robin ED (1987). Death by pulmonary artery flow-directed catheter (editorial). Time for a moratorium?. *Chest*.

[20] Connors AF, Speroff T, Dawson NV (1996). The effectiveness of right heart catheterization in the initial care of critically ill patients. *Journal of the American Medical Association*.

[21] Bernard GR, Sopko G, Cerra F (2000). Pulmonary artery catheterization and clinical outcomes: National Heart, Lung, and Blood Institute and Food and Drug Administration workshop report. *Journal of the American Medical Association*.

[22] Sandham JD, Hull RD, Frederick Brant R (2003). A randomized, controlled trial of the use of pulmonary-artery catheters in high-risk surgical patients. *New England Journal of Medicine*.

[23] Harvey S, Harrison DA, Singer M (2005). Assessment of the clinical effectiveness of pulmonary artery catheters in management of patients in intensive care (PAC-Man): a randomised controlled trial. *Lancet*.

[24] Yu DT, Platt R, Lanken PN (2003). Relationship of pulmonary artery catheter use to mortality and resource utilization in patients with severe sepsis. *Critical Care Medicine*.

[25] Wheeler AP, Bernard GR, Thompson BT (2006). Pulmonary-artery versus central venous catheter to guide treatment of acute lung injury. *New England Journal of Medicine*.

[26] Richard C, Warszawski J, Anguel N (2003). Early use of the pulmonary artery catheter and outcomes in patients with shock and acute respiratory distress syndrome: a randomized controlled trial. *Journal of the American Medical Association*.

[27] Resano FG, Kapetanakis EI, Hill PC, Haile E, Corso PJ (2006). Clinical outcomes of low-risk patients undergoing beating-heart surgery with or without pulmonary artery catheterization. *Journal of Cardiothoracic and Vascular Anesthesia*.

[28] Hill JA, Pauly DF, Olitsky DR (2005). Evaluation study of congestive heart failure and pulmonary artery catheterization effectiveness. *Journal of the American Medical Association*.

[29] Allen LA, Rogers JG, Warnica JW (2008). High mortality without ESCAPE: the registry of heart failure patients receiving pulmonary artery catheters without randomization. *Journal of Cardiac Failure*.

[30] Leier CV (2007). Invasive hemodynamic monitoring the aftermath of the ESCAPE trial. *Cardiology Clinics*.

[31] Chatterjee K (2009). The Swan-Ganz catheters: past, present, and future: a viewpoint. *Circulation*.

[32] Funk DJ, Moretti EW, Gan TJ (2009). Minimally invasive cardiac output monitoring in the perioperative setting. *Anesthesia and Analgesia*.

[33] Critchley LAH, Critchley JAJH (1999). A meta-analysis of studies using bias and precision statistics to compare cardiac output measurement techniques. *Journal of Clinical Monitoring and Computing*.

[34] Rhodes A, Sunderland R (2005). Arterial pulse power analysis: the LiDCOTM plus system. *Functional Hemodynamic Monitoring (Update in Intensive Care Medicine)*.

[35] Linton RAF, Band DM, Haire KM (1993). A new method of measuring cardiac output in man using lithium dilution. *British Journal of Anaesthesia*.

[36] Garcia-Rodriguez C, Pittman J, Cassell CH (2002). Lithium dilution cardiac output measurement: a clinical assessment of central venous and peripheral venous indicator injection. *Critical Care Medicine*.

[37] Mayer J, Boldt J, Poland R, Peterson A, Manecke GR (2009). Continuous arterial pressure waveform-based cardiac output using the FloTrac/Vigileo: a review and meta-analysis. *Journal of Cardiothoracic and Vascular Anesthesia*.

[38] Cecconi M, Dawson D, Grounds RM, Rhodes A (2009). Lithium dilution cardiac output measurement in the critically ill patient: determination of precision of the technique. *Intensive Care Medicine*.

[39] Sundar S, Panzica P (2010). LiDCO systems. *International Anesthesiology Clinics*.

[40] Jonas M (2008). Haemodynamic optimisation of the surgical patient revisited. *Anaesthesia International*.

[41] Hamilton TT, Huber LM, Jessen ME (2002). PulseCO: a less-invasive method to monitor cardiac output from arterial pressure after cardiac surgery. *Annals of Thoracic Surgery*.

[42] Cecconi M, Fawcett J, Grounds RM, Rhodes A (2008). A prospective study to evaluate the accuracy of pulse power analysis to monitor cardiac output in critically ill patients. *BMC Anesthesiology*.

[43] Kim JJ, Dreyer WJ, Chang AC, Breinholt JP, Grifka RG (2006). Arterial pulse wave analysis: an accurate means of determining cardiac output in children. *Pediatric Critical Care Medicine*.

[44] Missant C, Rex S, Wouters PF (2008). Accuracy of cardiac output measurements with pulse contour analysis (PulseCO*™*) and Doppler echocardiography during off-pump coronary artery bypass grafting. *European Journal of Anaesthesiology*.

[45] McCoy JV, Hollenberg SM, Dellinger RP (2009). Continuous cardiac index monitoring: a prospective observational study of agreement between a pulmonary artery catheter and a calibrated minimally invasive technique. *Resuscitation*.

[46] Yamashita K, Nishiyama T, Yokoyama T, Abe H, Manabe M (2007). Effects of vasodilation on cardiac output measured by PulseCO^*™*^. *Journal of Clinical Monitoring and Computing*.

[47] Costa MG, Della Rocca G, Chiarandini P (2008). Continuous and intermittent cardiac output measurement in hyperdynamic conditions: pulmonary artery catheter vs. lithium dilution technique. *Intensive Care Medicine*.

[48] Kim JJ, Dreyer WJ, Chang AC, Breinholt JP, Grifka RG (2006). Arterial pulse wave analysis: an accurate means of determining cardiac output in children. *Pediatric Critical Care Medicine*.

[49] Linton RA, Jonas MM, Tibby SM (2000). Cardiac output measured by lithium dilution and transpulmonary thermodilution in patients in a paediatric intensive care unit. *Intensive Care Medicine*.

[50] Pearse R, Dawson D, Fawcett J, Rhodes A, Grounds RM, Bennett ED (2005). Early goal-directed therapy after major surgery reduces complications and duration of hospital stay. A randomised, controlled trial [ISRCTN38797445]. *Critical Care*.

[51] Costa MG, Cecconi A, Sheju L, Chiarandini P, Pompei L, Della Rocca G (2009). Uncalibrated arterial pulse analysis cardiac output obtained with LiDCO Rapid versus PAC Thermodilution technique. *Intensive Care Medicine*.

[52] Abdel-Galil K, Craske D, McCaul J (2010). Optimisation of intraoperative haemodynamics: early experience of its use in major head and neck surgery. *British Journal of Oral and Maxillofacial Surgery*.

[53] Wijayasiri L, Garewal D, Khpal M, Rhodes A, Dewhurst A, Cecconi M (2010). Does stroke volume increase after a fluid challenge? A study on the management of patients undergoing major head and neck free flap surgery: preliminary data. *Critical Care*.

[54] Barbon E, Caliandro F, Kamdar J (2011). Dynamic indices of preload in postcardiac surgery patients by pulse power analysis. *Critical Care*.

[55] Pearse R Optimisation of peri-operative cardiovascular management to improve surgical outcome. http://public.ukcrn.org.uk/search/StudyDetail.aspx?StudyID=6307.

[56] Hofera CK, Cecconib M, Marxc G, Della Roccad G (2009). Minimally invasive haemodynamic monitoring. *European Journal of Anaesthesiology*.

[57] Wesseling KH, Purschke R, Smith NT (1976). A computer module for the continuous monitoring of cardiac output in the operating theatre and the ICU. *Acta Anaesthesiologica Belgica*.

[58] Mayer J, Suttner S (2009). Cardiac output derived from arterial pressure waveform. *Current Opinion in Anaesthesiology*.

[59] Hamzaoui O, Monnet X, Richard C, Osman D, Chemla D, Teboul JL (2008). Effects of changes in vascular tone on the agreement between pulse contour and transpulmonary thermodilution cardiac output measurements within an up to 6-hour calibration-free period. *Critical Care Medicine*.

[60] PULSION Medical Inc Training documents—advanced hemodynamic monitoring. http://www3.pulsion.de/fileadmin/pulsion_share/Education/Training/TraintheTrainer/TtT_MPI851405US_R00_101008_Parameters.pdf.

[61] Mathews L, Singh RK (2008). Cardiac output monitoring. *Annals of Cardiac Anaesthesia*.

[62] Buhre W, Weyland A, Kazmaier S (1999). Comparison of cardiac output assessed by pulse-contour analysis and thermodilution in patients undergoing minimally invasive direct coronary artery bypass grafting. *Journal of Cardiothoracic and Vascular Anesthesia*.

[63] Chakravarthy M, Patil TA, Jayaprakash K, Kalligudd P, Prabhakumar D, Jawali V (2007). Comparison of simultaneous estimation of cardiac output by four techniques in patients undergoing off-pump coronary artery bypass surgery–a prospective observational study. *Annals of Cardiac Anaesthesia*.

[64] Goedje O, Hoeke K, Lichtwarck-Aschoff M, Faltchauser A, Lamm P, Reichart B (1999). Continuous cardiac output by femoral arterial thermodilution calibrated pulse contour analysis: comparison with pulmonary arterial thermodilution. *Critical Care Medicine*.

[65] Wiesenack C, Prasser C, Keyl C, Rödig G (2001). Assessment of intrathoracic blood volume as an indicator of cardiac preload: single transpulmonary thermodilution technique versus assessment of pressure preload parameters derived from a pulmonary artery catheter. *Journal of Cardiothoracic and Vascular Anesthesia*.

[66] Peyton PJ, Chong SW (2010). Minimally invasive measurement of cardiac output during surgery and critical care: a meta-analysis of accuracy and precision. *Anesthesiology*.

[67] Halvorsen PS, Sokolov A, Cvancarova M, Hol PK, Lundblad R, Tønnessen TI (2007). Continuous cardiac output during off-pump coronary artery bypass surgery: pulse-contour analyses vs pulmonary artery thermodilution. *British Journal of Anaesthesia*.

[68] Della Rocca G, Costa MG, Coccia C (2003). Cardiac output monitoring: aortic transpulmonary thermodilution and pulse contour analysis agree with standard thermodilution methods in patients undergoing lung transplantation. *Canadian Journal of Anesthesia*.

[69] Boyle M, Lawrence J, Belessis A, Murgo M, Shehabi Y (2007). Comparison of dynamic measurements of pulse contour with pulsed heat continuous cardiac output in postoperative cardiac surgical patients. *Australian Critical Care*.

[70] Küntscher MV, Blome-Eberwein S, Pelzer M, Erdmann D, Germann G (2002). Transcardiopulmonary vs pulmonary arterial thermodilution methods for hemodynamic monitoring of burned patients. *Journal of Burn Care and Rehabilitation*.

[71] Hamm JB, Nguyen BV, Kiss G (2010). Assessment of a cardiac output device using arterial pulse waveform analysis, Vigileo*™*, in cardiac surgery compared to pulmonary arterial thermodilution. *Anaesthesia and Intensive Care*.

[72] Edwards Lifesciences LLC FloTrac system 3rd generation software: The next generation in hemodynamic management. http://www.edwards.com/sitecollectionimages/products/mininvasive/ar04099.pdf.

[73] De Backer D, Marx G, Tan A (2011). Arterial pressure-based cardiac output monitoring: a multicenter validation of the third-generation software in septic patients. *Intensive Care Medicine*.

[74] Compton FD, Zukunft B, Hoffmann C, Zidek W, Schaefer JH (2008). Performance of a minimally invasive uncalibrated cardiac output monitoring system (Flotrac*™*/Vigileo*™*) in haemodynamically unstable patients. *British Journal of Anaesthesia*.

[75] Mayer J, Boldt J, Beschmann R, Stephan A, Suttner S (2009). Uncalibrated arterial pressure waveform analysis for less-invasive cardiac output determination in obese patients undergoing cardiac surgery. *British Journal of Anaesthesia*.

[76] Compton F, Wittrock M, Schaefer J-H, Zidek W, Tepel M, Scholze A (2008). Noninvasive cardiac output determination using applanation tonometry-derived radial artery pulse contour analysis in critically ill patients. *Anesthesia and Analgesia*.

[77] Manecke GR, Auger WR (2007). Cardiac output determination from the arterial pressure wave: clinical testing of a novel algorithm that does not require calibration. *Journal of Cardiothoracic and Vascular Anesthesia*.

[78] McGee WT, Horswell JL, Calderon J (2007). Validation of a continuous, arterial pressure-based cardiac output measurement: a multicenter, prospective clinical trial. *Critical Care*.

[79] Biancofiore G, Critchley LAH, Lee A (2009). Evaluation of an uncalibrated arterial pulse contour cardiac output monitoring system in cirrhotic patients undergoing liver surgery. *British Journal of Anaesthesia*.

[80] Matthieu B, Karine N-G, Vincent C (2008). Cardiac output measurement in patients undergoing liver transplantation: pulmonary artery catheter versus uncalibrated arterial pressure waveform analysis. *Anesthesia and Analgesia*.

[81] Krejci V, Vannucci A, Abbas A, Chapman W, Kangrga IM (2010). Comparison of calibrated and uncalibrated arterial pressure-based cardiac output monitors during orthotopic liver transplantation. *Liver Transplantation*.

[82] Sakka SG, Kozieras J, Thuemer O, van Hout N (2007). Measurement of cardiac output: a comparison between transpulmonary thermodilution and uncalibrated pulse contour analysis. *British Journal of Anaesthesia*.

[83] Mayer J, Boldt J, Schöllhorn T, Röhm KD, Mengistu AM, Suttner S (2007). Semi-invasive monitoring of cardiac output by a new device using arterial pressure waveform analysis: a comparison with intermittent pulmonary artery thermodilution in patients undergoing cardiac surgery. *British Journal of Anaesthesia*.

[84] Mayer J, Boldt J, Wolf MW, Lang J, Suttner S (2008). Cardiac output derived from arterial pressure waveform analysis in patients undergoing cardiac surgery: validity of a second generation device. *Anesthesia and Analgesia*.

[85] Hofer CK, Senn A, Weibel L, Zollinger A (2008). Assessment of stroke volume variation for prediction of fluid responsiveness using the modified FloTrac^*™*^ and PiCCOplus^*™*^ system. *Critical Care*.

[86] Suehiro K, Okutani R (2010). Stroke volume variation as a predictor of fluid responsiveness in patients undergoing one-lung ventilation. *Journal of Cardiothoracic and Vascular Anesthesia*.

[87] Mayer J, Boldt J, Mengistu AM, Röhm KD, Suttner S (2010). Goal-directed intraoperative therapy based on autocalibrated arterial pressure waveform analysis reduces hospital stay in high-risk surgical patients: a randomized, controlled trial. *Critical Care*.

[88] Alhashemi JA, Cecconi M, Della Rocca G, Cannesson M, Hofer CK (2010). Minimally invasive monitoring of cardiac output in the cardiac surgery intensive care unit. *Current Heart Failure Reports*.

[89] Ng JM, Chow MY, Ip-Yam PC, Goh MH, Agasthian T (2007). Evaluation of partial carbon dioxide rebreathing cardiac output measurement during thoracic surgery. *Journal of Cardiothoracic and Vascular Anesthesia*.

[90] Kotake Y, Yamada T, Nagata H (2009). Improved accuracy of cardiac output estimation by the partial CO rebreathing method. *Journal of Clinical Monitoring and Computing*.

[91] Kotake Y, Moriyama K, Innami Y (2003). Performance of noninvasive partial CO_2_ rebreathing cardiac output and continuous thermodilution cardiac output in patients undergoing aortic reconstruction surgery. *Anesthesiology*.

[92] Gueret G, Kiss G, Rossignol B (2006). Cardiac output measurements in off-pump coronary surgery: comparison between NICO and the Swan-Ganz catheter. *European Journal of Anaesthesiology*.

[93] Botero M, Kirby D, Lobato EB, Staples ED, Gravenstein N (2004). Measurement of cardiac output before and after cardiopulmonary bypass: comparison among aortic transit-time ultrasound, thermodilution, and noninvasive partial CO_2_ rebreathing. *Journal of Cardiothoracic and Vascular Anesthesia*.

[94] Odenstedt H, Stenqvist O, Lundin S (2002). Clinical evaluation of a partial CO_2_ rebreathing technique for cardiac output monitoring in critically ill patients. *Acta Anaesthesiologica Scandinavica*.

[95] Mielck F, Buhre W, Hanekop G, Tirilomis T, Hilgers R, Sonntag H (2003). Comparison of continuous cardiac output measurements in patients after cardiac surgery. *Journal of Cardiothoracic and Vascular Anesthesia*.

[96] Van Heerden PV, Baker S, Lim SI, Weidman C, Bulsara M (2000). Clinical evaluation of the Non-invasive Cardiac Output (NICO) monitor in the intensive care unit. *Anaesthesia and Intensive Care*.

[97] Nilsson LB, Eldrup N, Berthelsen PG (2001). Lack of agreement between thermodilution and carbon dioxide-rebreathing cardiac output. *Acta Anaesthesiologica Scandinavica*.

[98] Rocco M, Spadetta G, Morelli A (2004). A comparative evaluation of thermodilution and partial CO_2_ rebreathing techniques for cardiac output assessment in critically ill patients during assisted ventilation. *Intensive Care Medicine*.

[99] Kubicek WG, Karnegis JN, Patterson RP, Witsoe DA, Mattson RH (1966). Development and evaluation of an impedance cardiac output system. *Aerospace Medicine*.

[100] Shoemaker WC, Wo CCJ, Bishop MH (1994). Multicenter trial of a new thoracic electrical bioimpedance device for cardiac output estimation. *Critical Care Medicine*.

[101] Lababidi Z, Ehmke DA, Durnin RE, Leaverton PE, Lauer RM (1970). The first derivative thoracic impedance cardiogram. *Circulation*.

[102] Shoemaker WC, Belzberg H, Wo CCJ (1998). Multicenter study of noninvasive monitoring systems as alternatives to invasive monitoring of acutely ill emergency patients. *Chest*.

[103] Sathyaprabha TN, Pradhan C, Rashmi G, Thennarasu K, Raju TR (2008). Noninvasive cardiac output measurement by transthoracic electrical bioimpedence: influence of age and gender. *Journal of Clinical Monitoring and Computing*.

[104] Gujjar AR, Muralidhar K, Banakal S, Gupta R, Sathyaprabha TN, Jairaj PS (2008). Non-invasive cardiac output by transthoracic electrical bioimpedence in post-cardiac surgery patients: comparison with thermodilution method. *Journal of Clinical Monitoring and Computing*.

[105] Barin E, Haryadi DG, Schookin SI (2000). Evaluation of a thoracic bioimpedance cardiac output monitor during cardiac catheterization. *Critical Care Medicine*.

[106] Appel PL, Kram HB, MacKabee J (1986). Comparison of measurements of cardiac output by bioimpedance and thermodilution in severely ill surgical patients. *Critical Care Medicine*.

[107] Clancy TV, Norman K, Reynolds R, Covington D, Maxwell JG (1991). Cardiac output measurement in critical care patients: thoracic electrical bioimpedance versus thermodilution. *Journal of Trauma*.

[108] Wong KL, Hou PC (1996). The accuracy of bioimpedance cardiography in the measurement of cardiac output in comparison with thermodilution method. *Acta Anaesthesiologica Sinica*.

[109] Van De Water JM, Miller TW, Vogel RL, Mount BE, Dalton ML (2003). Impedance cardiography the next vital sign technology?. *Chest*.

[110] Kööbi T, Kähönen M, Koskinen M, Kaukinen S, Turjanmaa VMH (2000). Comparison of bioimpedance and radioisotope methods in the estimation of extracellular water volume before and after coronary artery bypass grafting operation. *Clinical Physiology*.

[111] Spiess BD, Patel MA, Soltow LO, Wright IH (2001). Comparison of bioimpedance versus thermodilution cardiac output during cardiac surgery: evaluation of a second-generation bioimpedance device. *Journal of Cardiothoracic and Vascular Anesthesia*.

[112] Spinale FG, Reines HD, Crawford FA (1995). Comparison of bioimpedance and thermodilution methods for determining cardiac output: experimental and clinical studies: updated in 1995. *Annals of Thoracic Surgery*.

[113] Zácek P, Kunes P, Kobzová E, Dominik J (1999). Thoracic electrical bioimpedance versus thermodilution in patients post open-heart surgery. *Acta Medica (Hradec Králové)*.

[114] Hirschl MM, Kittler H, Woisetschläger C (2000). Simultaneous comparison of thoracic bioimpedance and arterial pulse waveform-derived cardiac output with thermodilution measurement. *Critical Care Medicine*.

[115] Keren H, Burkhoff D, Squara P (2007). Evaluation of a noninvasive continuous cardiac output monitoring system based on thoracic bioreactance. *American Journal of Physiology*.

[116] Rotcajg D, Denjean D, Estagnasie P, Brusset A, Squara P (2009). Comparison of monitoring performance of Bioreactance vs. pulse contour during lung recruitment maneuvers. *Critical Care*.

[117] Raval NY, Squara P, Cleman M, Yalamanchili K, Winklmaier M, Burkhoff D (2008). Multicenter evaluation of noninvasive cardiac output measurement by bioreactance technique. *Journal of Clinical Monitoring and Computing*.

[118] Marqué S, Cariou A, Chiche JD, Squara P (2009). Comparison between Flotrac-Vigileo and Bioreactance, a totally noninvasive method for cardiac output monitoring. *Critical Care*.

[119] CONMED Corporation ECOM endotracheal cardiac output monitor. http://www.conmed.com/products_ECOM.php.

[120] Wallace AW, Salahieh A, Lawrence A, Spector K, Owens C, Alonso D (2000). Endotracheal cardiac output monitor. *Anesthesiology*.

[121] Ball TR, Culp BC, Patel V, Gloyna DF, Ciceri DP, Culp Jr. WC (2010). Comparison of the endotracheal cardiac output monitor to thermodilution in cardiac surgery patients. *Journal of Cardiothoracic and Vascular Anesthesia*.

[122] Galstyan G, Bychinin M, Alexanyan M, Gorodetsky V (2010). Comparison of cardiac output and blood volumes in intrathoracic compartments measured by ultrasound dilution and transpulmonary thermodilution methods. *Intensive Care Medicine*.

[123] Krivitski NM, Kislukhin VV, Thuramalla NV (2008). Theory and in vitro validation of a new extracorporeal arteriovenous loop approach for hemodynamic assessment in pediatric and neonatal intensive care unit patients. *Pediatric Critical Care Medicine*.

[124] Tsutsui M, Matsuoka N, Ikeda T, Sanjo Y, Kazama T (2009). Comparison of a new cardiac output ultrasound dilution method with thermodilution technique in adult patients under general anesthesia. *Journal of Cardiothoracic and Vascular Anesthesia*.

[125] Savino JS, Troianos CA, Aukburg S, Weiss R, Reichek N (1991). Measurement of pulmonary blood flow with transesophageal two-dimensional and Doppler echocardiography. *Anesthesiology*.

[126] Perrino AC, Harris SN, Luther MA (1998). Intraoperative determination of cardiac output using multiplane transesophageal echocardiography: a comparison to thermodilution. *Anesthesiology*.

[127] Concha MR, Mertz VF, Cortínez LI, González KA, Butte JM (2009). Pulse contour analysis and transesophageal echocardiography: a comparison of measurements of cardiac output during laparoscopic colon surgery. *Anesthesia and Analgesia*.

[128] Berton C, Cholley B (2002). Equipment review: new techniques for cardiac output measurement—oesophageal Doppler, Fick principle using carbon dioxide, and pulse contour analysis. *Critical Care*.

[129] Dicorte CJ, Latham P, Greilich PE, Cooley MV, Grayburn PA, Jessen ME (2000). Esophageal doppler monitor determinations of cardiac output and preload during cardiac operations. *Annals of Thoracic Surgery*.

[130] Madan AK, UyBarreta VV, Aliabadi-Wahle S (1999). Esophageal doppler ultrasound monitor versus pulmonary artery catheter in the hemodynamic management of critically III surgical patients. *Journal of Trauma*.

[131] Mythen MG, Webb AR (1995). Perioperative plasma volume expansion reduces the incidence of gut mucosal hypoperfusion during cardiac surgery. *Archives of Surgery*.

[132] Sinclair S, James S, Singer M (1997). Intraoperative intravascular volume optimisation and length of hospital stay after repair of proximal femoral fracture: randomised controlled trial. *British Medical Journal*.

[133] Laupland KB, Bands CJ (2002). Utility of esophageal Doppler as a minimally invasive hemodynamic monitor: a review. *Canadian Journal of Anesthesia*.

[134] Monnet X, Chemla D, Osman D (2007). Measuring aortic diameter improves accuracy of esophageal Doppler in assessing fluid responsiveness. *Critical Care Medicine*.

[135] Bundgaard-Nielsen M, Holte K, Secher NH, Kehlet H (2007). Monitoring of peri-operative fluid administration by individualized goal-directed therapy: review article. *Acta Anaesthesiologica Scandinavica*.

[136] Singer M (2006). The FTc is not an accurate marker of left ventricular preload. *Intensive Care Medicine*.

[137] Schober P, Loer SA, Schwarte LA (2009). Perioperative hemodynamic monitoring with transesophageal doppler technology. *Anesthesia and Analgesia*.

[138] Singer M (1998). Cardiac output in 1998. *Heart*.

[139] Dark PM, Singer M (2004). The validity of trans-esophageal Doppler ultrasonography as a measure of cardiac output in critically ill adults. *Intensive Care Medicine*.

[140] Boulnois JLG, Pechoux T (2000). Non-invasive cardiac output monitoring by aortic blood flow measurement with the Dynemo 3000. *Journal of Clinical Monitoring and Computing*.

[141] Venn R, Steele A, Richardson P, Poloniecki J, Grounds M, Newman P (2002). Randomized controlled trial to investigate influence of the fluid challenge on duration of hospital stay and perioperative morbidity in patients with hip fractures. *British Journal of Anaesthesia*.

[142] Gan TJ, Soppitt A, Maroof M (2002). Goal-directed intraoperative fluid administration reduces length of hospital stay after major surgery. *Anesthesiology*.

[143] Wakeling HG, McFall MR, Jenkins CS (2005). Intraoperative oesophageal Doppler guided fluid management shortens postoperative hospital stay after major bowel surgery. *British Journal of Anaesthesia*.

[144] Noblett SE, Snowden CP, Shenton BK, Horgan AF (2006). Randomized clinical trial assessing the effect of Doppler-optimized fluid management on outcome after elective colorectal resection. *British Journal of Surgery*.

[145] Conway DH, Mayall R, Abdul-Latif MS, Gilligan S, Tackaberry C (2002). Randomised controlled trial investigating the influence of intravenous fluid titration using oesophageal Doppler monitoring during bowel surgery. *Anaesthesia*.

[146] Chytra I, Pradl R, Bosman R, Pelnář P, Kasal E, Židková A (2007). Esophageal Doppler-guided fluid management decreases blood lactate levels in multiple-trauma patients: a randomized controlled trial. *Critical Care*.

[147] McKendry M, McGloin H, Saberi D, Caudwell L, Brady AR, Singer M (2004). Randomised controlled trial assessing the impact of a nurse delivered, flow monitored protocol for optimisation of circulatory status after cardiac surgery. *British Medical Journal*.

